# Mr. Philip Kuhles

**Published:** 1912-03

**Authors:** 


MR. PHILIP KUHLES, who has had the longest service in pharmacy in western New York, died in his home at Lackawana, Feb. 12, 1912, aged 67.

